# Is Cycling Practice Related to Men’s Pelvic Floor Dysfunctions? A Hypothesis-Generating Observational Study

**DOI:** 10.3390/ijerph18041923

**Published:** 2021-02-17

**Authors:** Guadalupe Molina-Torres, Mirari Ochandorena-Acha, Yune Echazarreta-Aparicio, Nuria Sánchez-Labraca, Manuel González-Sánchez, Marc Terradas-Monllor, Luz A. Varela-Vásquez, Jose Antonio Merchán-Baeza, Eduard Minobes-Molina

**Affiliations:** 1 Department of Nursing Science, Physiotherapy and Medicine, Faculty of Health Sciences, University of Almeria, 04120 Almeria, Spain; gmt940@ual.es (G.M.-T.); yune.ea@gmail.com (Y.E.-A.); msl397@ual.es (N.S.-L.); 2 Research Group on Methodology, Methods, Models and Outcomes of Health and Social Sciences (M3O), Faculty of Health Sciences and Welfare, University of Vic-Central University of Catalonia (UVIC-UCC), C. Sagrada Familia, 7, 08500 Vic, Spain; mirari.ochandorena@uvic.cat (M.O.-A.); marc.terradas@uvic.cat (M.T.-M.); luzadriana.varela@uvic.cat (L.A.V.-V.); eduard.minobes@uvic.cat (E.M.-M.); 3 Department of Psychiatry and Physiotherapy, Institute of Biomedical Research of Malaga (IBIMA), University of Malaga, 29071 Malaga, Spain; mgsa23@uma.es

**Keywords:** pelvic floor, urinary incontinence, erectile dysfunction, cycling

## Abstract

Background: There is a lack of consensus with regards to the consequences of cycling practice on urogenital and sexual problems in men. The aim of the study was to analyse the relationship between intensity of cycling practice and urinary tract symptoms, erectile dysfunction, and urinary incontinence. Methods: Observational hypothesis-generating design. Cyclists, men, between 25 and 70 years who had been cycling for more than one year were included. During the statistical analysis, a multiple linear regression model, partial correlation and Spearman’s correlation were carried out. Results: Fifty-eight men participated in the study. Results showed that there is a correlation between years of cycling and prostate symptoms (*p* = 0.041), and between age and erectile dysfunction (*p* = 0.001). The multiple linear regression model and the partial correlation analysis showed a correlation between the years of cycling and prostate symptoms (*p* = 0.007 and *p* = 0.018). Conclusions: The results have shown that there is a slight correlation between the years of cycling and the presence of lower urinary tract symptoms, independently of the man’s age. Therefore, the results display that high-intensity cycling practice might impact negatively in some men’s pelvic floor functions. Further research is needed to analyse the impact of cycling on urogenital problems in this population group.

## 1. Introduction

The malfunction of the set of muscles that comprises the pelvic floor can generate various affectations that constitute a health problem for millions of people, affecting their quality of life [1]. Some of these disorders are related to overactive bladder problems, urinary incontinence, perineal pain, and sexual dysfunction [1]. The prevalence in the Spanish population of overactive bladder and urinary incontinence in men aged 50–65 years is around 5%, while erectile dysfunction in adults aged 25–70 years is 18.9% [2,3,4]. These affectations can be influenced by various health factors, such as hypertension, obesity, diabetes, increasing age, as well as high-impact exercise [5].

Regarding the exercise, a common feature is that flexion activities of the hip, such as climbing, squatting, cycling, provoke or worsen urogenital pain. Many of these patients played American football, lifted weights, or wrestled as teenagers and young adults, or are cyclists [6]. Recent scientific evidence links the practice of both professional and recreational cycling with pelvic dysfunction in men [7]. Although cycling offers widely known health benefits, some studies report that, in addition to the common wrist, knee, or gluteal injuries, cycling appears to have a negative influence on men’s sexual and urinary function [8,9,10].

This negative influence is associated with the constant compression of the perineum against the saddle during cycling, causing perineal injuries such as ulcerations and sores on the skin, calluses and the formation of fibrous masses on the ischial tuberosities which, in turn, can cause perineal pain [10]. The compression of the pudendal and cavernous nerve can lead to impotence due to numbness in the penis and scrotum, which can remit in weeks or persist for months [11,12]. Cyclists should be vigilant of pain or numbness in specific genital regions, as reduced sensation or discomfort in these locations is associated with increased risk of erectile dysfunction [13]. At the urinary level, traumatic events of the urethra due to compression that can cause dysuria in males have also been reported [10]. Less common problems associated with cycling include priapism, penile thrombosis, infertility, spermatic cord torsion, and elevated prostate antigen [8].

Recent studies establish cycling as an important risk factor in pelvic floor dysfunction and its consequences [11,12], being a major healthcare burden heavily affecting patients’ Quality of Life [6]. These findings have generated growing interest in the association between cycling and urogenital problems in men, both recreationally and professionally [14,15]. In the scientific literature, different profiles of cyclists have been determined, considering the time and intensity of training. For example, Awad et al. considered high-intensity practice to be that carried out by cyclists who had been training for more than two years, three or more times a week, for more than 25 miles [7]. Low-intensity practice was any practice that did not meet these parameters [7]. However, in another study, the division was made between cyclists with moderate practice, training less than three hours per day, and professional cyclists, training more than three hours per day [16].

Therefore, there is variability between studies in relation to the classification of cycling based on time or intensity, in the same way that the consequences of cycling at the urinary and sexual level in men have not been agreed upon [7,17]. For these reasons, the present study aimed to analyse the possible relationship between the years and weekly hours of cycling with lower urinary tract symptoms (LUTS), erectile dysfunction (ED) and urinary incontinence (UI) in male cyclists. Thus, we hypothesized that high-intensity (>10 years or >10 h/week) cycling practice negatively impacts men’s pelvic floor functions.

## 2. Materials and Methods 

### 2.1. Study Design

The study used a transversal, exploratory and observational hypothesis-generating cohort design in accordance with the Strengthening the Reporting of Observational Studies in Epidemiology (STROBE) [18].

### 2.2. Ethical Aspects

This study complies with the ethical criteria defined in the Declaration of Helsinki (2014) and the current European legislation regarding confidentiality and data protection [19]. During the development of the study, Organic Law 3/2018, of 5 December, on the Protection of Personal Data and Guarantee of Digital Rights was complied with. All participants signed an informed consent. The Bioethics Commission of the University of Almería approved the project (UALBIO2018/009). 

### 2.3. Settings and Data Collection 

The recruitment process and data collection were carried out at the University of Almería (Spain) by three physiotherapists during the period between February and June 2019.

### 2.4. Participants

Every volunteer from the provinces of Almería and Granada (Spain) enrolled during the recruitment period was screened for eligibility. Male cyclists, aged between 25 and 75, who had been practicing cycling for more than one year, were eligible for the study. Exclusion criteria were: men who had previously undergone a prostatectomy, and those who used erection-stimulating drugs.

### 2.5. Outcome Variables and Measurements

The sociodemographic variables collected were age, weight, height, and body mass index (BMI). Information regarding the years of sports practice and the hours of practice per week was also collected. 

In addition, the following self-administered and anonymised questionnaires were used:

#### 2.5.1. The International Scale of Prostate Symptoms (IPSS)

The IPSS is a self-administered questionnaire that assesses the severity of lower urinary tract symptoms (LUTS) associated with benign prostatic hyperplasia (BPH). It consists of 6 items that must be scored from 0 to 5 (0 being none and 5 almost always). The interpretation of the results is carried out as follows: a total score of 1–7 is considered mild symptomatology, 8–19 moderate, and 20–35 severe. The Spanish version of the questionnaire is valid, reliable (alpha = 0.79), and sensitive to clinical changes (effect size= 2.52) and has demonstrated psychometric properties equivalent to the original instrument [20].

#### 2.5.2. The International Index Erectile Function (IIEF-5)

The IIEF-5 is a multidimensional, self-administered questionnaire that assesses male sexual function. It consists of 15 items with 5 response options (0 being none and 5 always). The results are interpreted as follows: a score of 5–7, severe ED; 8–11, moderate ED; 12–16, mild to moderate ED; 17–21, mild ED; >22 does not suffer from ED. It is considered a simple, easy-to-use and reliable instrument (alpha = 0.92), validated for the Spanish population [21].

#### 2.5.3. The International Consultation on Incontinence Questionnaire—Short Form (ICIQ-SF)

This self-administered questionnaire identifies people with urinary incontinence and its impact on the quality of life. It consists of 4 questions for rating of symptoms in the past 4 weeks. The sum score of the first 3 questions must be added to obtain the total score. The final question is a self-diagnostic item that is unscored. In the interpretation of the results, any score higher than zero is considered a diagnosis of UI. This questionnaire is considered a reliable instrument (alpha = 0.89) and it has satisfactory psychometric properties, so its use is recommended in clinical practice [22].

### 2.6. Study Size

Participants’ recruitment was carried out between the months of February and May 2019 in a consecutive and non-random way. The number of participants recruited during this period determined the total number of the sample, which was 58 participants.

### 2.7. Statistical Analysis

Statistical analysis was performed using the programme Statistical Package for the Social Sciences, version 27 (SPSS Inc., Chicago, IL, USA).

Regarding the cycling practice intensity, the variable corresponding to the years of cycling practice was categorised into “less than 10 years of practice” and “more than 10 years of practice”. Also, the variable related to weekly cycling hours was also categorised into “less than 10 h a week” and “more than 10 h a week”.

In the descriptive analysis of the characteristics of the sample, the means, and standard deviations, for continuous variables, frequencies and percentages were calculated. Subsequently, an analysis was carried out between cycling (hours/week and years of practice) and pelvic symptoms (prostate symptoms, urinary incontinence, and erectile dysfunction) using cross tables, obtaining the frequencies and percentages.

To study whether the variables followed a normal distribution, the Kolmogorov–Smirnov test was used. Since the variables did not follow a normal distribution, the Spearman correlation test was used to study the relationship between demographic variables of cyclists and cycling with the presence of pelvic symptoms.

In the case of the IPSS and IIEF-5, the raw values were used for the correlation analysis. To determine the size of the effect of the correlations, the coefficients (ρ) between 0 and 0.39 were considered to be slight correlations; between 0.40 and 0.69 moderate; and between 0.70 and 0.89 strong [23].

To examine the capacity of baseline measurements to predict prostatic symptoms (IPSS), a multiple linear regression model was used. The strength of the association was examined using regression coefficients (B), R^2^, adjusted R^2^ and *p*-values. In addition, standardised beta coefficients (b) were included for all predictors in the final model to allow direct comparisons between predictor variables in the regression model. In order to guarantee the validity of the regression model, the assumptions of normality, linearity, autocorrelation and no multi-collinearity were analysed. For this purpose, standardised residuals were calculated, and Kolmogorov–Smirnov analysis was carried out to assess normal distribution of the residuals. Also, casewise diagnostics were performed to identify outlier values (those that were more than three standard deviations from the regression line). Subsequently, Cook’s distances (*D*) and leverage values were used to identify the influence of those outliers in the regression model. Leverage values were considered influential if > than 2(*k + 1)*/n, where *k* is the number of the explanatory variables in the model and n is the sample size, and Cook’s distances (*D*) if *D* > 1. Finally, the Durbin–Watson test was used to assess autocorrelation of residuals from the regression analysis (values around 2 indicate the independence of residuals) and variance inflation factors were computed to quantify multi-collinearity.

In addition, partial correlation analysis was used to assess the association between years of cycling and lower urinary tract symptoms controlling for the age of the participants.

For all the analyses, *p*-values of less than 0.05 were considered statistically significant results.

## 3. Results

### 3.1. Descriptive Results

A total of 58 subjects were screened for eligibility. All of them met the established criteria and agreed to participate in the study. The mean age of the participants was 45.72 (9.72) years, with a mean weight and height of 77.45 (10.46) kg and 176 (6.54) cm, 60.3% of the participants had a normal BMI. Of note, 55.2% of the participants had been cycling for more than 10 years, and 62.1% did so for less than 10 h a week; 63.8% of the participants showed mild prostate symptoms, 84.5% did not suffer from urinary incontinence and 81% did not suffer from erectile dysfunction. The descriptive analysis of the relation between the years of practice and the hours of weekly practice based on the pelvic symptoms did not show significant findings (Table 1 and Table 2). In turn, it was observed that, of the cyclists who had been cycling for less than 10 years (n = 26), 69.2% showed mild prostate symptoms, 88.5% did not suffer from urinary incontinence and 84.6% did not suffer from erectile dysfunction. In the cyclists who accumulated more than 10 years of practice (n = 32), it was identified that 59.4% suffered mild prostate symptoms, 81.2% did not present urinary incontinence and 78.1% did not suffer from erectile dysfunction (Table 1). Regarding the weekly practice hours, it was observed that, of the cyclists who practiced less than 10 h a week (n = 36), 69.4% showed mild prostate symptoms, 83.3% did not suffer from urinary incontinence and 80.6% did not suffer from dysfunction erectile. In the cyclists who practiced more than 10 h a week (n = 22), it was identified that 54.5% suffered mild prostate symptoms, 86.4% did not present urinary incontinence and 81.8% did not suffer from erectile dysfunction (Table 2).

### 3.2. Correlations between Variables

The results obtained during the correlation analysis can be seen in Table 3. The Kolmogorov–Smirnov test showed the non-normal distribution of the variables. The correlation analysis between demographic variables and pelvic symptoms revealed that there is a slight correlation between years of cycling and prostate symptoms (*p* = 0.270, *p* = 0.041), and a moderate correlation between age and erectile dysfunction (*p* = −0.416, *p* = 0.001). Finally, erectile dysfunction showed a slight correlation with prostate symptoms (*p* = −0.279, *p* = 0.034).

### 3.3. Multiple Linear Regression

In the multiple linear regression model, the raw values of the IPSS were included as a dependent variable, as it was the only one that showed some correlation with the variables corresponding to cycling. The only variable that remained in the model was related to the years of cycling, explaining the variance of 11% (R^2^ = 0.112, adj R^2^ = 0.096). The non-autocorrelation and non-multicollinearity assumptions were met. However, the standardised residuals did not show a normal distribution (*p* = 0.007) and the diagnosis by cases identified an outlier. The Cook distance (0.185) and the leverage value (0.014) showed that this value was not influential in the model. The results are shown in Table 4.

### 3.4. Partial Correlations

The partial correlation analysis between the years of cycling and prostate symptoms, controlling for the age of the participants, revealed that there was a significant correlation between both variables regardless of the age of the participants (R = 0.312, *p* = 0.018).

## 4. Discussion

Cycling is becoming increasingly popular as a mode of transportation and exercise. In relation to health, cycling improves both cardiorespiratory fitness and cardiovascular risk factors [24], but it also involves health risks, most commonly traumatic and overuse injuries, [25] as well as concerns for various genitourinary conditions [26]. Although anecdotal evidence indicates a high prevalence of pelvic floor dysfunction, more specific studies are still scarce.

The present research aimed to examine the possible relationship between years and weekly hours of cycling practice with LUTS, ED, and UI in male cyclists.

In relation to the first finding, there is a slight correlation between those riders who had cycled for more than 10 years and the presence of LUTS. Some authors have found that cycling seems to have no statistically measurable effect on LUTS in amateur recreational cyclists [7]. However, the sample of this study was a total of 22 amateur male cyclists who had cycled for a period of 1 to 10 years. Therefore, the results in the present study might suggest a correlation between the presence of LUTS and those subjects with more than 10 years of practice. Moreover, it has been ruled out by partial correlation analysis that these results could be influenced by the age of the participants, reinforcing the idea that years of cycling are related to the presence of LUTS.

The results concerning erectile dysfunction indicate that there is no correlation between cycling and ED. In the literature, there is conflicting evidence regarding the relationship between cycling and ED, and many of the studies are limited in statistical power [12]. Nevertheless, Gan et al. published a recent systematic review and meta-analysis, the former using only validated metrics of ED, in which they reported that, when comparing cyclists to non-cyclists in an unadjusted analysis, there were no significant differences in the odds of having ED [27]. These conclusions are consistent with the findings in the present article. On the other hand, various authors have indicated that the type of saddle used, as well as the position of the rider, has more effect on resultant sexual dysfunction than simply participation in cycling [13,28]. Therefore, future research should include and consider these results. 

Another finding of the present study is a slight correlation between the presence of ED and LUTS. The available literature documents results confirming those of the present study: the prevalence of ED in patients with LUTS is high, and the occurrence of ED is significantly correlated with the degree of LUTS [29]. However, there is a lack of studies that examine these results specifically in cyclists. 

Regarding the UI results, no association was observed between UI and years of cycling, or hours of practice per week. A recent study aimed to determine the prevalence of UI among elite athletes and, to compare prevalence between sexes and across different sports modalities, reported that in male athletes, no correlation was found between UI and training volume or frequency [30]. The modality showing the greatest prevalence of UI was rugby [30]. This sport is characterised by repeated bursts of high-intensity exercise, running spurts and bruising [31], increasing forces directed toward the pelvic floor. In line with our findings, no incidence of UI was observed for cycling. This could be due to the nature of the intensity of exercise, movements, and impact on the ground. In rugby players, the main cause is thought to be a rise in abdominal pressure followed by abdominal muscle contraction in the absence of prior pelvic muscle contraction [32], and this is not shown in cyclists.

Regarding the clinical significance of the research, it has been proven that high-intensity cycling practice seems to negatively impact men’s pelvic floor functions. Some strategies to prevent these consequences could be to correctly adjust the characteristics of the bicycle, the position on the saddle, as well as the position of the rider [28]. In addition, cyclists should be vigilant of pain or numbness in specific anatomic regions such as the penis or perineum, as discomfort in these locations was associated with increased risk of pelvic floor dysfunctions [13]. The present study has several limitations that should be considered when interpreting the results. It has been performed as a hypothesis-generating study, as no statistical plan was provided during the protocol design process. Therefore, no sample size calculation was conducted. The small sample size might be another weakness, since it reduces the power of the study’s findings. During the statistical analysis, no relevant multiplicity adjustments were carried out, so the results must be interpreted prudently. The statistical results suggested that the residuals were not normally distributed and, although an outlier was detected, the statistical tests showed that it was not influential as it had no significant effect on the regression equation. Collectively, the displayed results should be considered with caution, and future research should consider these limitations. A study design with larger samples would allow for creation of subgroups according to mechanical factors (saddle designs, position of the riders, type of bikes or styles of cycling), facilitating more definitive conclusions regarding these factors.

## 5. Conclusions

In summary, the present study has shown that there is a slight correlation between those male cyclists who had cycled for more than 10 years and the presence of LUTS, independently of age. Therefore, this study adds to the literature the finding that high-intensity cycling practice seems to impact negatively in some men’s pelvic floor functions. Further research is needed to develop some strategies for the prevention of this cyclist profile, taking into account mechanical factors, such as the position of the rider or the type of saddle used.

## Figures and Tables

**Table 1:** Cross tables with years of practice and pelvic symptoms.

	Years of Cycling Practice		
Variables	Less than 10 Years (n = 26)	More than 10 Years (n = 32)	*p-*Value
Prostate symptoms (IPSS)			0.085 ^a^
No symptomatology	4 (15.4)	1 (3.1)	
Mild symptomatology	18 (69.2)	19 (59.4)	
Moderate symptomatology	4 (15.4)	8 (25.0)	
Severe symptomatology	0 (0)	4 (12.5)	
Urinary incontinence (ICIQ-SF)			0.495 ^b^
Yes	3 (11.5)	6 (18.8)	
No	23 (88.5)	26 (81.2)	
Erectile dysfunction (IIEF-5)			0.764 ^a^
No dysfunction	22 (84.6)	25 (78.1)	
Mild dysfunction	2 (7.7)	4 (12.5)	
Mild–moderate dysfunction	1 (3.8)	1 (3.1)	
Moderate dysfunction	0 (0)	2 (6.3)	
Severe dysfunction	1 (3.8)	0 (0)	

ICIQ-SF: The International Consultation on Incontinence Questionnaire–Short-Form; IIEF-5: The International Index Erectile Function; IPSS: The International Scale of Prostate Symptoms. ^a^ linear by linear association; ^b^ Fisher’s exact test.

**Table 2:** Cross tables with hours of practice and pelvic symptoms.

	Hours of Cycling/Week		
Variables	Less than 10 h/w (n = 36)	More than 10 h/w (n = 22)	*p-*Value
Prostate symptoms (IPSS)			0.103 ^a^
No symptomatology	4 (11.1)	1 (4.5)	
Mild symptomatology	25 (69.4)	12 (54.5)	
Moderate symptomatology	5 (13.9)	7 (31.8)	
Severe symptomatology	2 (5.6)	2 (9.1)	
Urinary incontinence (ICIQ-SF)			0.534 ^b^
Yes	6 (16.7)	3 (13.6)	
No	30 (83.3)	19 (86.4)	
Erectile dysfunction (IIEF-5)			0.652 ^a^
No dysfunction	29 (80.6)	18 (81.8)	
Mild dysfunction	5 (13.9)	1 (4.5)	
Mild–moderate dysfunction	1 (2.8)	1 (4.5)	
Moderate dysfunction	0 (0)	2 (9.1)	
Severe dysfunction	1 (2.8)	0 (0)	

h/w: hours per week; ICIQ-SF: The International Consultation on Incontinence Questionnaire-Short-Form; IIEF-5: The International Index Erectile Function; IPSS: The International Scale of Prostate Symptoms. ^a^ linear by linear association; ^b^ Fisher’s exact test.

**Table 3:** Spearman correlation analysis.

Spearman Correlation	IPSS (Total Score)	ICIQ-SF	IIEF-5 (Total Score)
Age	0.109	0.050	−0.416 **^,b^
Body mass index	0.048	0.015	0.197
Years of practice	0.270 *^,a^	0.099	−0.177
Hours of practice per week	0.206	−0.076	−0.019
IPSS (total score)	-	0.210	−0.279 *
ICIQ-SF	0.210	-	−0.123
IIEF-5 (total score)	−0.279 *	−0.123	-

* The correlation is significant at the 0.05 level; ** the correlation is significant at the 0.01 level. ^a^ mild correlation; ^b^ moderate correlation. ICIQ-SF: International Consultation on Incontinence Questionnaire-Short-Form; IIEF-5: International Index Erectile Function; IPSS: International Prostate Symptom Scale.

**Table 4:** Multiple linear regression model.

Predictor Variables	Regression Coefficient (B)	Standard Error	Standardised Coefficient (β)	*p*-Value	VIF
Age	-	-	-	0.23	1.19
Years of practice	4.33	1.63	0.33	0.01	1.00
Hours of practice/week	-	.	-	0.36	1.21

Overall model: R^2^ = 0.112, adjusted R^2^ = 0.096, F = 7.04, Std. residuals mean (SD) = 0.00 (0.99), Z = 0.139, *p* = 0.007, Durbin–Watson = 1.42.

## Data Availability

The data presented in this study are available on request from the corresponding author.
